# Standardization of D2 lymphadenectomy and surgical quality control (KLASS-02-QC): a prospective, observational, multicenter study [NCT01283893]

**DOI:** 10.1186/1471-2407-14-209

**Published:** 2014-03-19

**Authors:** Hyoung-Il Kim, Hoon Hur, Youn Nam Kim, Hyuk-Joon Lee, Min-Chan Kim, Sang-Uk Han, Woo Jin Hyung

**Affiliations:** 1 Department of Surgery, Yonsei University College of Medicine, 50 Yonsei-ro, Seodaemun-gu, Seoul 120-752, Korea; 2 Robot and MIS Center, Severance Hospital, Yonsei University Health System, 50 Yonsei-ro, Seodaemun-gu, Seoul 120-752, Korea; 3 Department of Surgery, Ajou University School of Medicine, 206 World Cup-ro, Yeongtong-gu, Suwon 443-749, Korea; 4 Department of Biostatistics, Yonsei University College of Medicine, 50 Yonsei-ro, Seodaemun-gu, Seoul 120-752, Korea; 5 Department of Surgery, Seoul National University College of Medicine, 101 Daehang-ro, Jongno-gu, Seoul 110-744, Korea; 6 Department of Surgery, Minimally Invasive and Robot Center, Dong-A University College of Medicine, 3-1 Dongdaeshin-dong, Seo-gu, Busan 602-715, Korea

**Keywords:** Neoplasms of stomach, D2 lymphadenectomy, Gastrectomy, Laparoscopy, Standardization, Quality control

## Abstract

**Background:**

Extended systemic lymphadenectomy (D2) is standard procedure for surgical treatment of advanced gastric cancer (AGC) although less extensive lymphadenectomy (D1) can be applied to early gastric cancer. Complete D2 lymphadenectomy is the mandatory procedure for studies that evaluate surgical treatment results of AGC. However, the actual extent of D2 lymphadenectomy varies among surgeons because of a lacking consensus on the anatomical definition of each lymph node station. This study is aimed to develop a consensus for D2 lymphadenectomy and also to qualify surgeons that can perform both laparoscopic and open D2 gastrectomy.

**Methods/Design:**

This (KLASS-02-QC) is a prospective, observational, multicenter study to qualify the surgeons that will participate in the KLASS-02-RCT, which is a prospective, randomized, clinical trial comparing laparoscopic and open gastrectomy for AGC. Surgeons and reviewers participating in the study will be required to complete a questionnaire detailing their professional experience and specific gastrectomy surgical background/training, and the gastrectomy metrics of their primary hospitals. All surgeons must submit three laparoscopic and three open D2 gastrectomy videos, respectively. Each video will be allocated to five peer reviewers; thus each surgeon’s operations will be assessed by a total of 30 reviews. Based on blinded assessment of unedited videos by experts’ review, a separate review evaluation committee will decide whether or not the evaluated surgeon will participate in the KLASS-02-RCT. The primary outcome measure is each surgeon’s proficiency, as assessed by the reviewers based on evaluation criteria for completeness of D2 lymphadenectomy.

**Discussion:**

We believe that our study for standardization of D2 lymphadenectomy and surgical quality control (KLASS-02-QC) will guarantee successful implementation of the subsequent KLASS-02-RCT study. After making consensus on D2 lymphadenectomy, we developed evaluation criteria for completeness of D2 lymphadenectomy. We also developed a unique surgical standardization and quality control system that consists of recording unedited surgical videos, and expert review according to evaluation criteria for completeness of D2 lymphadenectomy. We hope our systematic approach will set a milestone in surgical standardization that is essential for surgical clinical trials. Additionally, our methods will serve as a novel system for educating surgeons and assessing surgical proficiency.

**Trial registration:**

NCT01283893.

## Background

Laparoscopic gastrectomy is gaining wide acceptance for treating early gastric cancer because of its favorable short-term outcomes compared to open gastrectomy, including reduced blood loss, less pain, and faster recovery [1,2]. In addition, long-term outcomes following laparoscopic gastrectomy are comparable to conventional open gastrectomy for early gastric cancer [3,4]. Consequently, laparoscopic gastrectomy is now also considered for treating advanced gastric cancer, to provide the potential benefits of a minimally invasive surgical solution to this disease [5,6].

For surgical treatment of advanced gastric cancer, gastrectomy with D2 lymphadenectomy is recommended as a standard procedure in major guidelines because D2 lymphadenectomy results in better patient survival than D1 lymphadenectomy [7-11]. Thus, for studies that evaluate the surgical treatment results of advanced gastric cancer, complete D2 lymphadenectomy is a mandatory procedure. However, D2 dissection is known to be a technically challenging surgical procedure and dissection quality and completeness varies among surgeons. The actual extent of D2 lymphadenectomy varies among surgeons because there remains a lack of consensus on the anatomical definition of appropriate lymph node dissection extent reflected in ambiguous definitions with the published documentation [12]. Despite great efforts to enhance quality control in a previous study that compared D1 versus D2 lymph node dissection, inadequate removal of lymph node station(s) for complete D2 lymphadenectomy, i.e., the non-compliance rate, was reported to be 81.0% [13]. Considering the high rate of inadequate D2 lymphadenectomy in open surgery, even worse results would be anticipated in laparoscopic surgery because of its increased technical difficulty. To get accurate results in clinical trials of laparoscopic and open D2 lymphadenectomy for advanced gastric cancer, only surgeons who can perform exact D2 lymphadenectomy should participate, to ensure objective comparisons.

As far as we know, there has been no system to objectively evaluate the gastrectomy procedure focused on D2 lymphadenectomy quality. Furthermore, no study has yet attempted to standardize D2 lymphadenectomy during laparoscopic or open gastrectomy for clinical trials. Therefore, a tool that can assess the surgical proficiency and quality of lymphadenectomy is necessary. Herein, we report the development of a new surgical standardization and quality control system for assessing D2 lymphadenectomy. We intend to use this system to optimize our planned randomized prospective clinical trial to compare laparoscopic D2 lymphadenectomy with open D2 lymphadenectomy (KLASS-02-RCT, NCT01456598), to clarify the surgical feasibility and oncological safety of laparoscopic gastrectomy for advanced gastric cancer. Before initiating the prospective RCT, we conducted this quality control study (KLASS-02-QC, NCT01283893) to make a consensus for D2 lymphadenectomy and to qualify surgeons that can perform both laparoscopic and open D2 gastrectomy.

## Methods

### Study design

**Figure 1:**
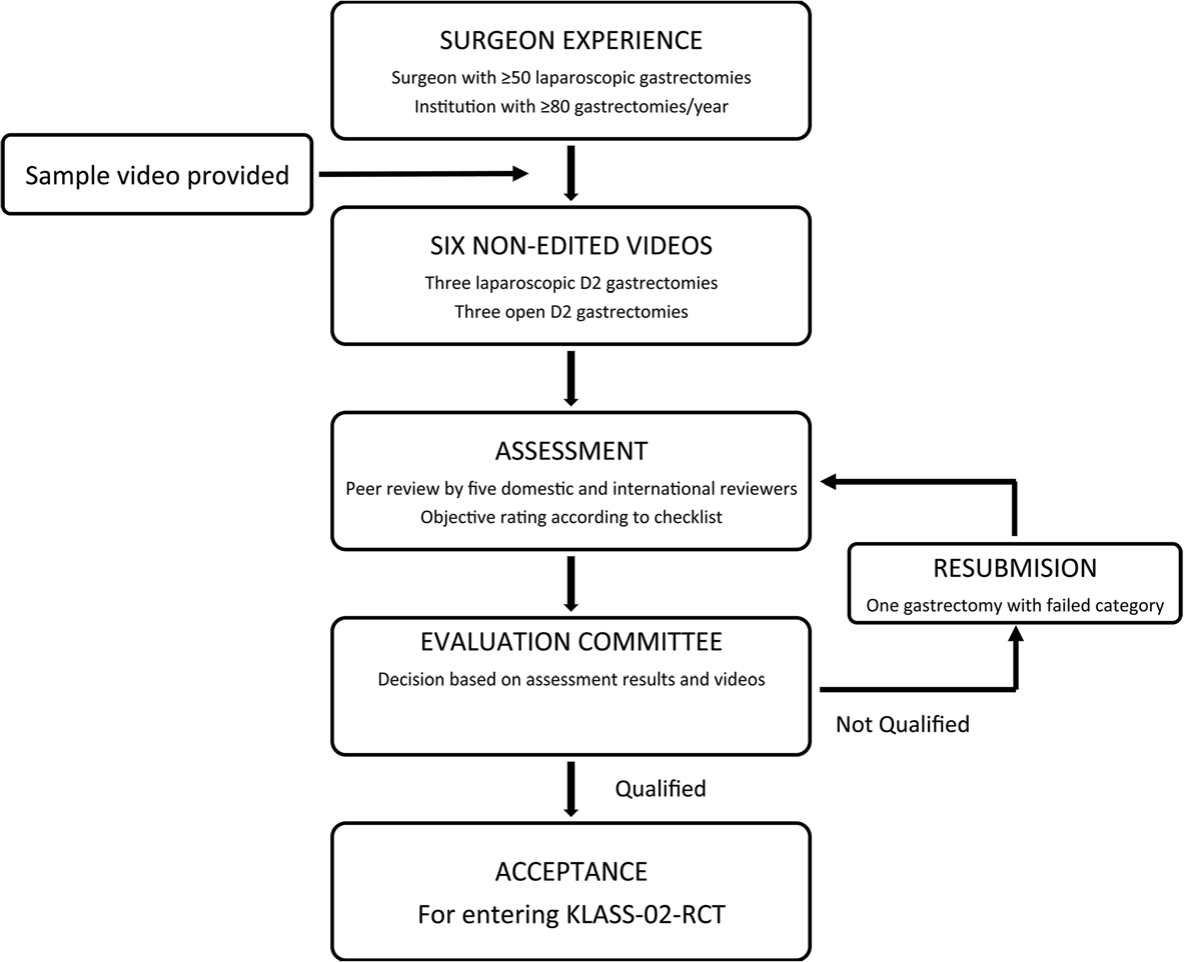
Study schema.

This quality control study (KLASS-02-QC) is a prospective, observational, multicenter study to assess surgeon competency in performing laparoscopic and open D2 lymphadenectomy (Figure 1). Assessments are based on expert rating of unedited surgical videos, according to evaluation criteria for completeness of D2 lymphadenectomy (Additional file 1: Table S1). Only qualified surgeons will participate in the planned clinical trial comparing laparoscopic and open gastrectomy for treating advanced gastric cancer (KLASS-02-RCT). This study was reviewed and approved by institute review board of Severance hospital (4-2010-0637).

### Study objectives

#### Primary aim

To qualify the surgeons participating in the clinical trial (KLASS-02-RCT), which will compare laparoscopic versus open gastrectomy, with D2 lymphadenectomy.

#### Secondary aims

1) To determine the feasibility of surgical standardization based on evaluation criteria for completeness of D2 lymphadenectomy; 2) To evaluate potential surgical proficiency improvement due to the review process; and 3) To assess potential relationships among the rated score, surgeon professional background, and the perioperative surgical outcomes of the patients whose operations were recorded.

### Organization

#### Data center

The role of the data center is to recruit participating surgeons and reviewers; to get signed non-disclosure agreements and completed questionnaires of recruited surgeons (Additional file 2: Table S2) and reviewers (Additional file 3: Table S3); to support video recording services for open gastrectomy cases; to blind the information regarding the patients, surgeons, and reviewers; to distribute submitted surgical videos; to collect reviewer assessments of surgical videos; and to disseminate committee decisions on the surgeons that are selected for participation in the subsequent RCT.

#### Reviewers

Korean and international surgeons will be invited as reviewers of surgical video recordings. To participate as a reviewer, experts will be required to submit a signed non-disclosure agreement and complete a reviewer questionnaire (Additional file 3: Table S3), which includes information such as personal gastrectomy experience and patient volume at their primary working hospital.

#### Review evaluation committee

The review evaluation committee consists of experts in laparoscopic and open D2 lymphadenectomy, including invited surgeon members of the Korean Gastric Cancer Association and principal investigators of the KLASS-02-RCT. Members of the committee developed a consensus on the mandatory anatomical extent of lymph node dissection, evaluation criteria for completeness of D2 lymphadenectomy, and sample surgical videos were then devoted based on this consensus. The review evaluation committee is distinct from the reviewer group, and will be the final determiner of a surgeon’s technical competency and approval for participation in the KLASS-02-RCT, based on the reviewers’ assessments and surgical videos in a blinded way.

### Study process

#### Evaluation criteria for completeness of D2 lymphadenectomy

Definition of D1 lymphadenectomy is removal of the perigastric lymph nodes. Definition of D2 lymphadenectomy is additional removal of a second tier of lymph nodes in the extraperigastric areas, which generally fall along branches of the celiac axis including the left gastric, splenic, common hepatic, and proper hepatic arteries. The assessment form consists of 22 elements of D2 lymphadenectomy that together evaluate the success or failure to perform a surgical task (Additional file 1: Table S1). Although the assessment form was made based on the anatomical definition of lymph node stations, [12] parameters for assessing patient safety and surgical quality are also included in the criteria.

#### Sample videos of D2 lymphadenectomy

The review evaluation committee made unedited and edited sample videos of laparoscopic and open gastrectomy for surgeons, which satisfy the evaluation criteria for completeness of D2 lymphadenectomy. The sample videos clearly show the D2 lymphadenectomy, a total omentectomy with lymph node dissection of stations including 1, 3, 4, 5, 6, 7, 8a, 9, 11p and 12a, based on Japanese classifications are performed with appropriate annotations [12].

#### Recruitment of reviewers and surgeons

Surgeon recruitment will be continued until 484 patients have been enrolled in KLASS-02-RCT, at which point planned analysis for safety related to morbidity and mortality is planned.

##### Reviewers

Thirty reviewers are required to fully assess the surgical videos of participating surgeons in this quality control study. Thus, our target reviewer enrollment number is 50, which provide a sizeable margin to account for potential drop-outs and work overload.

##### Surgeons

The data center recruits surgeons who perform open and laparoscopic gastrectomy in clinical practice. After public offering of study participation to the members of the Korean Gastric Cancer Association, candidates will be accepted who satisfy following criteria: Surgeons who have personally performed more than 50 cases of each gastrectomy approach (i.e., laparoscopic and open), and surgeons that work in institutions where more than 80 gastrectomies are performed annually. To participate, the surgeon must obtain approval required by the Institutional Review Board of each institute, and should submit a completed surgeon questionnaire (Additional file 2: Table S2). This questionnaire includes surgeon information such as personal gastrectomy experience, annual case volume at their institute, and whether their institute has a specialized multidisciplinary team for gastric cancer treatment.

#### Recording unedited surgical videos

**Criteria of patients for video recording:** Patients whose operation will be recorded are required to provide written informed consent. Patients with mental incompetency, are illiterate, pregnant, or are <20 years old or >80 years old will not be asked to participate in this study. Patients must have documented biopsy-proven gastric cancer without distant lymph node metastasis or plans for combined operations. While all resectable gastric cancer patients without adjacent organ invasion are candidates for an open gastrectomy procedure, patients who will undergo a laparoscopic surgery must have gastric cancer without evidence of serosal invasion and extra-perigatric lymph node metastasis, as determined by evaluation of preoperative CT-scans, upper endoscopy, or endoscopic ultrasound.

**Video recording:** Recommended surgical procedures are same as those provided in the sample videos. Reconstruction methods, surgical instruments for anastomosis, and drainage insertion are in accordance with the surgeon’s preference. Video recording should clearly identify the extent of lymph node dissection and should record the entire procedure, without edition. No identifiable information of the patient or surgeon should be recorded.

#### Video submissions and assessment

Unedited videos of three laparoscopic and three open gastrectomies must be submitted by each participating surgeon to the data center. At the same time, case report forms (CRFs) containing perioperative surgical outcomes also will be submitted. The data center will blind the surgeon information and five reviewers will be randomly allocated to evaluate each video. Reviewers will assess each video based on the evaluation criteria for completeness of D2 lymphadenectomy, and will also give general impression and comments regarding surgeon performance.

#### Decision-making on surgeon qualification

The review evaluation committee will make a decision on surgeon qualification to participate in the KLASS-02-RCT based on the reviewers’ assessments. Additional review of surgical video could be necessary for selected cases. All CRFs containing perioperative surgical outcome will be blinded to preclude potential reviewer and committee bias. Evaluation committee decisions will be categorized as: (1) Qualified; the surgeon’s operation proficiency is sufficient to accept them as qualified to participate in the KLASS-02-RCT; (2) Resubmission Required; when the surgeon performance in the submitted operation videos of either or both gastrectomy methods are not satisfactory, the evaluation committee will ask for resubmission of a video and CRF for the failed approach(es); and (3) Not-Qualified; surgeons whose operation performance is insufficient, and must be evaluated by submitting another six videos after getting re-approval from their Institutional Review Board. If a surgeon fails to be deemed qualified even after video resubmission, that surgeon will be regarded as Not-Qualified and will not participate in the RCT.

#### Timeline

All prospective RCT surgeons must participate in this quality control trial and only qualified surgeons will participate in the subsequent RCT. All surgeons must submit all required surgical videos and CRFs containing perioperative surgical outcome within 6 months after IRB approval. Otherwise, the surgeon will be regarded as a study dropout. The reviewer assessment process will be completed within 1 month after submission of required videos and CRFs. The review evaluation committee will make decisions after completing the review process for every five sequentially evaluated surgeons.

#### Statistical considerations: estimated number of surgeons, video recordings, reviewers, and assessments

This study is not a hypothesis-testing trial and therefore does not include an accepted approach for power calculation. Lacking previous reports on the criteria for surgical competency, we estimated the number of surgical videos and reviewers that are sufficient to assess surgeon proficiency, within realistic ranges. An overall target of six videos for each surgeon and five independent reviewers for each video were set. Considering patient variation, three operations for each surgical approach would be an acceptable number for surgeon assessment. Considering the number of expert surgeons at hospitals with appropriate volume, the estimated number of participating surgeons in Korea is approximately 50. Each surgeon must submit the minimum of six videos (three for each procedure), and up to eight videos if required for re-evaluation. The estimated total number of videos will range from 300 to 350. Thus, final estimated target patient enrollment for gastrectomy video recording will number 350.

#### Analysis plan

To explore qualified surgeon characteristics, descriptive statistical methods without formal testing will be used. For evaluating the secondary measurements, data will be analyzed using both quantitative and qualitative techniques. For assessing inter-reviewer agreement on video ratings, a generalized Kappa statistic will be determined. The major factor of the qualification decision, i.e., the average reviewer score based on predetermined criteria for submitted operation videos, will be used for the quantitative evaluation of the secondary measurements. Relationships among the surgeon proficiency rating scores, background information of the participating surgeons and reviewers, decision results by the review evaluation committee, and perioperative surgical outcomes will be analyzed. Data will be analyzed using SAS 9.2 software (SAS Institute Inc., Cary, NC, USA). For all statistical purposes, p < 0.05 will be considered indicative of statistically significant differences.

## Discussion

We believe that effectiveness and safety of D2 dissection in advanced gastric cancer can be evaluated only when the D2 surgical procedure is standardized and surgical quality is controlled. If the surgery as an intervention in a clinical trial is of variable quality, the results will not be helpful to identify accurate differences in surgical outcomes using different specific treatments. Such variability would undermine the study findings. Despite attempting quality control of lymph node dissection, the results of a previous study comparing D1 versus D2 lymphadenectomy were weakened because of surgeon non-compliance (inadequate removal of lymph node stations) and contamination (lymph nodes were detected outside the intended level of dissection) rates of 81.0% and 27.1%, respectively [13]. Furthermore, the morbidity and mortality of the D2 group were 43% and 10%, respectively [14]. To compare the surgical outcome of laparoscopic and open D2 lymphadenectomy, lymph node dissection should be a homogenous surgical intervention. We designed this study protocol to assess the proficiency of a surgeon who performs both laparoscopic and open D2 lymphadenectomy. To assure that the standardized procedure is performed, we developed a standardization and quality control protocol.

For an RCT comparing two surgical approaches for lymphadenectomy suffers from either high inter-surgeon or intra-surgeon performance variability, this will adversely affect the power to identify significant differences in outcomes between the two treatments. Thus, we prepared sample videos for surgeons to demonstrate essential principles of lymphadenectomy that must be maintained during surgery. Sample videos with annotations were made for easier understanding of the anatomical dissection of each lymph node station. Video demonstration is markedly superior to merely reading the written definitions of the appropriate anatomical extent of lymph node station excision during gastrectomy. We believe that distributing these sample videos will help to implement the consensus by the review evaluation committee on the mandatory anatomical extent of lymph node dissection required, and performed by surgeons participating in the study.

To control surgical quality, we suggest assessment using unedited surgical video as assessment and learning tools. Unedited videos can clearly show not only the lymphadenectomy extent but also adverse events that may happen during surgery, such as injuries to adjacent organs and accidental bleeding. Any event that can jeopardize patient safety would be identified by reviewing unedited videos. In addition, using video clips can help peer reviewers to assess the proficiency of a surgeon without personally visiting the operating theater. Removing this limitation will increase the efficiency of the review process because we can provide more robust assessment of surgical proficiency by recruiting a larger number of reviewers.

To objectively assess the surgeon’s proficiency, we prepared an assessment form consisting of 22 evaluation criteria for completeness of D2 lymphadenectomy. By analyzing inter-reviewer agreement based on the background information of the reviewer, we expect to determine the required number of surgical videos and reviewers required for optimal assessment. Furthermore, our system provides feedback to each surgeon in the form of proficiency assessment score and allow for surgeon learning and improvement if they fail to initially qualify, by requesting resubmission of additional unedited videos. We will be able to evaluate whether the resubmission process improves the surgical proficiency of a surgeon. If this is the case, our method of standardization and quality control can be used for educating novice surgeons, as well as experienced surgeons that are learning new surgical techniques.

Developing a quality control consensus for D2 lymphadenectomy performance during gastrectomy, recording unedited surgical videos, and assessing surgeon proficiency according to evaluation criteria for completeness of D2 lymphadenectomy may provide important benefits to clinical practice. Laparoscopic D2 lymphadenectomy for advanced gastric cancer itself was criticized for its applicability because of its inherent technical difficulty. If laparoscopic D2 lymphadenectomy benefits patients, it should not be discarded because of technical difficulty; instead, solutions should be sought to improve technique performance. We conceived this study as one solution, by implementing a system for surgical standardization and quality control during gastrectomy. This method will provide important new information and education to improve patient surgical outcomes when treating gastric cancer with minimally invasive laparoscopic approaches.

In conclusion, we believe that our study for standardization of D2 lymphadenectomy and surgical quality control (KLASS-02-QC) will guarantee successful implementation of the subsequent clinical trial comparing laparoscopic and open D2 lymphadenectomy for advanced gastric cancer (KLASS-02-RCT). After making consensus on D2 lymphadenectomy, we developed evaluation criteria for completeness of D2 lymphadenectomy. We also developed a unique surgical standardization and quality control system that consists of recording unedited surgical videos, and expert review according to evaluation criteria for completeness of D2 lymphadenectomy. We hope that our systematic approach will set a milestone in surgical standardization that is essential for surgical clinical trials. Additionally, our methods will serve as a novel system for educating surgeons and for assessing surgical proficiency.

## Abbreviations

AGC: Advanced gastric cancer; KLASS: Korean Laparoscopic Gastrointestinal Surgery Study Group; CRF: Case report form; RCT: Randomized clinical trial; QC: Quality control.

## Competing interests

All authors declare that they have no competing financial interests to declare.

## Authors’ contributions

KHI drafted the manuscript and is the primary author of this manuscript. HH, LHJ, and KMC designed this study and participated in the developing the D2 lymphadenectomy consensus. KYN helped draft the manuscript, provided statistical counseling in clinical trial design, and will conduct the primary statistical analyses. HSU is the grant holder, and conceived and initiated the study design. HWJ conceived and initiated the study design, and also supervised the manuscript construction. All authors have read and approved the final manuscript for publication.

## Pre-publication history

The pre-publication history for this paper can be accessed here:

http://www.biomedcentral.com/1471-2407/14/209/prepub

## Supplementary Material

Additional file 1: Table S1 Evaluation criteria for completeness of subtotal D2 lymphadenectomy. Click here for file

Additional file 2: Table S2 Questionnaire for surgeons. Click here for file

Additional file 3: Table S3 Questionnaire for reviewers. Click here for file
