# Supplementary material for: Standardization of D2 lymphadenectomy and surgical quality control (KLASS-02-QC): a prospective, observational, multicenter study [NCT01283893]
Source: BMC Cancer. 2014 Mar 19;14:209. doi: 10.1186/1471-2407-14-209 (PMC4000001; doi:10.1186/1471-2407-14-209)
Supplement: Additional file 1: Table S1 — Evaluation criteria for completeness of subtotal D2 lymphadenectomy. [file 1471-2407-14-209-S1.doc]

**Table S1**. Evaluation criteria for completeness of subtotal D2 lymphadenectomy

| **Surgical Video Assessment Form**  Video ID: ___________________________ Reviewer ID: _________________________________  Please indicate whether or not the surgeon meets the requirements of the D2 lymph node dissection in the video you are reviewing by encircling either “yes” or “no” next to the 22 defined elements of the procedure. In cases where the surgeon fails to perform the required dissection, please identify the reason the requirement was not met.   | **Procedure** | **Station** | **Requirement** | **Meets the requirement** | **In case of “No” please identify the reason** | | --- | --- | --- | --- | --- | | 1. Total omentectomy |  | No injury was made to the any other organ. | Yes / No |  | | 2. Division of left gastroepiploic artery  (It is not necessary to dissect the root of left gastroepiploic artery if the tumor is located in lower third of the stomach.) | 4Sb | The left gastroepiploic artery and left gastroepiploic vein are divided at least below the bifurcation of the first gastric branch. | Yes / No |  | |  | No injury was made to the colon of splenic flexure. | Yes / No |  | | 4d | The branch of right gastroepiploic artery and vein are retrieved. | Yes / No |  | | 3. Appropriate extent of No. 6 lymph node (LN) dissection | 6 | The right gastroepiploic vein is divided just above the bifurcation of the anterior superior pancreaticoduodenal vein and the right gastroepiploic vein. | Yes / No |  | |  |  | The right gastroepiploic artery is divided just peripheral to the bifurcation of the right gastroepiploic artery and the anterior superior pancreaticoduodenal artery. | Yes / No |  | |  |  | The lowest anterior superior pancreaticoduodenal vein is identified and exposed. | Yes / No |  | |  |  | The prepancreatic soft tissues above the lowest anterior superior pancreaticoduodenal vein are completely removed. | Yes / No |  | |  |  | The prepancreatic soft tissues above the level of the bifurcation of the anterior superior pancreaticoduodenal vein and right gastroepiploic vein are completely removed. | Yes / No |  | |  |  | No injury was made to the pancreatic parenchyma. | Yes / No |  | | 4. Appropriate extent of No. 5 LN dissection | 5 | The root of right gastric artery is identified and exposed. | Yes / No |  | | 5. Appropriate extent of No. 12a LN dissection | 12a | The lower half of the proper hepatic artery is exposed; at least its anterior and left surfaces. | Yes / No |  | |  |  | The left side of the portal vein is identified and exposed and soft tissues are completely removed. | Yes / No |  | | 6 .Appropriate extent of No. 8a LN dissection | 8a | The common hepatic artery is exposed; at least its anterior and superior surfaces. | Yes / No |  | |  |  | The soft tissues above the upper edge of the pancreas are completely removed. | Yes / No |  | | 7. Appropriate extent of No. 9 LN dissection  (resection of the celiac plexus is not necessary) | 9 | The retroperitoneal membrane is divided along the boundary between the right crus and the soft tissues around the celiac trunk to completely dissect No. 9 LNs. | Yes / No |  | | 8. Appropriate extent of No. 7 LN dissection | 7 | The root of the left gastric artery is exposed and ligated. | Yes / No |  | | 9.Appropriate extent of No. 11p LN dissection | 11p | The proximal half of the splenic artery is exposed, from its root to the site where the meandering splenic artery is in the closest vicinity to the stomach. | Yes / No |  | |  |  | The splenic vein is identified and exposed, or at least the dorsal side of pancreatic parenchyma is exposed. | Yes / No |  | | 10.Prevention of pancreatic injury during suprapancreatic LN dissection |  | No pancreatic injury by heat of energy devices and/or assistant’s forceps was caused. | Yes / No |  | | 11.Appropriate extent of No. 1 and 3 LN dissection | 1, 3 | The soft tissue attached to the lesser curvature side of gastric wall is completely removed. | Yes / No |  | |  |  | No esophageal and/or gastric injury by heat of energy devices and/or blind manipulation was caused. | Yes / No |  |   In the following space provided, please give us your general impressions and comments about the D2 LN dissection performed in the video. You may attach additional sheets, if necessary.   | General impression and comments: | | --- | |
| --- | --- | --- | --- | --- | --- | --- | --- | --- | --- | --- | --- | --- | --- | --- | --- | --- | --- | --- | --- | --- | --- | --- | --- | --- | --- | --- | --- | --- | --- | --- | --- | --- | --- | --- | --- | --- | --- | --- | --- | --- | --- | --- | --- | --- | --- | --- | --- | --- | --- | --- | --- | --- | --- | --- | --- | --- | --- | --- | --- | --- | --- | --- | --- | --- | --- | --- | --- | --- | --- | --- | --- | --- | --- | --- | --- | --- | --- | --- | --- | --- | --- | --- | --- | --- | --- | --- | --- | --- | --- | --- | --- | --- | --- | --- | --- | --- | --- | --- | --- | --- | --- | --- | --- | --- | --- | --- | --- | --- | --- | --- | --- | --- | --- | --- |
