# Supplementary material for: Standardization of D2 lymphadenectomy and surgical quality control (KLASS-02-QC): a prospective, observational, multicenter study [NCT01283893]
Source: BMC Cancer. 2014 Mar 19;14:209. doi: 10.1186/1471-2407-14-209 (PMC4000001; doi:10.1186/1471-2407-14-209)
Supplement: Additional file 2: Table S2 — Questionnaire for surgeons. [file 1471-2407-14-209-S2.doc]

**Table S2**. Questionnaire for surgeons

| **A. Participating surgeon Information**   1. ***Background***    1. When did you get your accreditation for your subspecialty (year)?    2. How many years of fellowship training do you have?    3. Do you have fellowship training in UGI or gastric surgery? If yes, how long did you train? (months) 2. ***Experience***    1. How many gastrectomies did you assist with before starting your own surgery?       1. Open gastrectomies ( ) cases       2. Laparoscopic gastrectomies ( ) cases    2. How many gastrectomies did you performed for life?       1. Open gastrectomies ( ) cases       2. Laparoscopic gastrectomies ( ) cases 3. ***Case numbers***    1. How many gastric cancer operations do you perform per month (excluding reoperations for complications)?       1. Open gastrectomies ( ) cases/month       2. Laparoscopic gastrectomies ( ) cases/month    2. How many gastric cancer operations did you performed last year?       1. Open gastrectomies ( ) cases       2. Laparoscopic gastrectomies ( ) cases   **B. Hospital Information**   1. How many gastrectomies were performed in your hospital last year (excluding reoperations for complications)?    1. Open gastrectomies ( ) cases    2. Laparoscopic gastrectomies ( ) cases 2. Is your hospital a tertiary center?    1. Yes    2. No 3. Does your hospital have residents?    1. Yes    2. No 4. Number of in-patient beds    1. Less than 500    2. 500-1000 beds    3. More than 1000 5. Total number of operating rooms in your hospital?    1. Less than 10    2. 10-30    3. More than 30 6. Do you have dedicated laparoscopic surgery operating rooms?    1. Yes    2. No 7. What type of laparoscopic system(s) do you use? –Name of company ( )    1. HD monitors    2. LCD monitors    3. Straight scopes    4. Flexible scopes 8. Do you have a Surgical ICU?    1. YES    2. NO 9. Do you have dedicated personnel (or nurses) that manage perioperative patient care?    1. YES    2. NO 10. Who assists you in the surgery?     1. Another attending/staff surgeon     2. Fellows ( )     3. Physician’s assistant( )     4. Residents ( )        1. If resident, what year in training? ( ) yr 11. Does your institution hold multidisciplinary gastric cancer-related conferences?     1. YES     2. NO 12. Does your institution have a dedicated GI radiologist(s)?     1. YES     2. NO 13. Does your institution have a dedicated pathologist(s)?     1. YES     2. NO |
| --- |
