# Supplementary material for: Standardization of D2 lymphadenectomy and surgical quality control (KLASS-02-QC): a prospective, observational, multicenter study [NCT01283893]
Source: BMC Cancer. 2014 Mar 19;14:209. doi: 10.1186/1471-2407-14-209 (PMC4000001; doi:10.1186/1471-2407-14-209)
Supplement: Additional file 3: Table S3 — Questionnaire for reviewers. [file 1471-2407-14-209-S3.doc]

**Table S3. Questionnaire for reviewers**

| **A. Reviewer Information**   1. Background    1. In what year did you get accreditation for your subspecialty (year)?    2. How many years of fellowship training have you completed?    3. Do you have fellowship training in UGI or gastric surgery? If yes, how long did you train (months)? 2. Experience    1. How many gastrectomies did you assist with before starting your own surgery?       1. Open gastrectomies ( ) cases       2. Laparoscopic gastrectomies ( ) cases    2. How many total gastrectomies have you performed in your life?       1. Open gastrectomies ( ) cases       2. Laparoscopic gastrectomies ( ) cases 3. Case numbers    1. How many gastric cancer operations do you perform per month (excluding reoperations for complications)?       1. Open gastrectomies ( ) cases/month       2. Laparoscopic gastrectomies ( ) cases/month    2. How many gastric cancer operations did you perform last year?       1. Open gastrectomies ( ) cases       2. Laparoscopic gastrectomies ( ) cases   **B. Hospital Information**   1. How many gastrectomies were performed in your hospital last year (excluding reoperations for complications)?    1. Open gastrectomies ( ) cases    2. Laparoscopic gastrectomies ( ) cases 2. Is your hospital a tertiary center?    1. Yes    2. No 3. Does your hospital have resident physicians?    1. Yes    2. No 4. Number of in-patient beds?    1. Less than 500    2. 500-1000 beds    3. More than 1000 5. Total number of operating rooms in your hospital?    1. Less than 10    2. 10-30    3. More than 30 6. Do you have dedicated laparoscopic surgery operating rooms?    1. Yes    2. No 7. What type of laparoscopic system(s) do you use? –Name of company ( )    1. HD monitors    2. LCD monitors    3. Straight scopes    4. Flexible scopes 8. Who assists you in the surgery?    1. Another attending/staff surgeon    2. Fellows ( )    3. Physician’s assistant( )    4. Residents ( )       1. If resident, what year in training? ( ) yr |
| --- |
